# Effectiveness of Hand Washing Education on Knowledge, Attitude and Practice among Orang Asli Kids in Gua Musang, Kelantan

**DOI:** 10.21315/mjms-01-2025-059

**Published:** 2025-10-31

**Authors:** Ling Huey Khong, Anis Kausar Ghazali, Nik Zuraina Nik Mohd Noor, Nur Rina Alissa Razian, Anis Amiera Muhamad Alojid, Wan Alif Syazwani Wan Alias, Fauziah Ismail, Nor Reah Mustafa, Cik Siti Nuraini Abdullah, Sharizan Awang, Wan Noor Faradila Aini Mat Latif, Tuan Noorkorina Tuan Kub

**Affiliations:** 1 Department of Medical Microbiology and Parasitology, School of Medical Sciences, Health Campus, Universiti Sains Malaysia, Kubang Kerian, Kelantan, Malaysia; 2 School of Health Sciences, Health Campus, Universiti Sains Malaysia, Kubang Kerian, Kelantan, Malaysia; 3 Biostatistics and Research Methodology Unit, School of Medical Sciences, Health Campus, Universiti Sains Malaysia, Kubang Kerian, Kelantan, Malaysia; 4 Medical Microbiology and Parasitology Laboratory, Hospital Pakar Universiti Sains Malaysia, Health Campus, Universiti Sains Malaysia, Kubang Kerian, Kelantan, Malaysia; 5 Infection Control and Hospital Epidemiology Unit, Hospital Pakar Universiti Sains Malaysia, Health Campus, Universiti Sains Malaysia, Kubang Kerian, Kelantan, Malaysia

**Keywords:** handwashing, knowledge, attitudes, practices, Orang Asli

## Abstract

**Background:**

Handwashing is a crucial preventive measure against illnesses such as diarrhoea and respiratory infections, particularly in rural communities with limited access to clean water and sanitation. Orang Asli kids often experience high rates of preventable diseases due to poor handwashing practices. This study aimed to determine the knowledge, attitudes, and practices (KAP) of handwashing among Orang Asli kids in Gua Musang, Kelantan, and to assess the impact of a targeted handwashing education intervention.

**Methods:**

An interventional study was conducted among 61 Orang Asli students from Sekolah Kebangsaan Pasir Linggi, Gua Musang, Kelantan. The intervention consisted of a structured handwashing education programme, and KAP scores were measured before and after the intervention. A paired sample t-test was used to analyse the impact of the intervention on the participants’ KAP scores.

**Results:**

The post-intervention scores for all KAP related questions were significantly higher than the pre-intervention scores (*P* < 0.05). Following the educational programme, there was a significant improvement in handwashing with soap, which had been poorly practised before the intervention. Year 1 students had lower baseline knowledge and attitude scores compared to older students, highlighting the need for greater focus on younger children in future programmes.

**Conclusion:**

The handwashing education programme effectively improved the KAP of handwashing among Orang Asli kids. However, the lower baseline KAP scores among younger students suggested that future interventions should focus more on early education to foster better hygiene practices and prevent the spread of infectious diseases in this vulnerable community.

## Introduction

Handwashing is a core hand hygiene practice that involves the use of plain or antimicrobial soap and water to physically remove dirt, destroy transient microorganisms, and reduce resident flora (1). Hands often serve as an essential vector in many infectious diseases, such as gastrointestinal infections like diarrhoea and respiratory illnesses like pneumonia. Therefore, handwashing is considered one of the most efficient and cost-effective approaches to breaking the chain of disease transmission, thereby reducing disease occurrence (2).

Promoting handwashing enhances basic sanitation and prevents the spread of infectious diseases among populations, particularly in developing countries. Nonetheless, on a global scale, around three in ten individuals, or 2.3 billion people, lack access to handwashing facilities with water and soap in their homes (3). The lack of handwashing facilities poses a significant challenge in maintaining proper sanitation and hygiene, which potentially leads to adverse health effects, especially in children. Globally, diarrhoeal diseases are one of the leading causes of death among the child population aged 0 to 14 years, responsible for one in every 10 deaths within this age group (4). In fact, childhood diarrhoea is found to be significantly associated with handwashing without using soap (5). Moreover, respiratory infections are not only the leading cause of infectious disease burden worldwide but also responsible for half of all child deaths annually (6).

Access to hygiene infrastructure is often worse in rural areas. Individuals residing in rural regions frequently face greater challenges in accessing basic handwashing facilities than those living in urban areas (7). In Malaysia, the Aboriginal people, also known as “Orang Asli,” predominantly live in rural and remote areas. They are often disadvantaged in terms of socioeconomic status, education, and health. Consequently, the prevalence of diseases among the Orang Asli community, particularly among kids, is greater compared to the general population (8). Most diseases occurring among Aboriginal kids are attributed to poor handwashing practices.

One of the key barriers to effective handwashing among Orang Asli kids is a lack of knowledge, appropriate attitudes, and consistent practices. Research has consistently shown that handwashing education can significantly reduce the incidence of infectious diseases and contribute to maintaining school health (9). Proper handwashing also reduces school absenteeism among children, which indirectly improves their academic performance and eventually contributes to the nation’s development (10). Therefore, healthcare providers and researchers should conduct comprehensive and sustained handwashing training programmes, especially in schools serving vulnerable populations, such as the Orang Asli (11). This is particularly important because Orang Asli kids are more susceptible to infectious diseases due to overcrowded environments and limited access to basic handwashing facilities (7, 12).

Primary school age is a critical developmental stage for forming lasting personal hygiene habits. At this stage, children are more open to adopting new behaviours, making it an ideal period for intervention. Handwashing programmes specifically designed for young school-aged children have been shown to produce positive results in terms of both behaviour and health outcomes (13, 14). Despite these findings, most existing studies have focused on children living in urban areas in Malaysia or Aboriginal communities in other countries. As a result, there is a lack of available literature regarding the KAP of handwashing among Orang Asli kids in Malaysia.

In light of this context, this study aims to evaluate the Aboriginal kids’ KAP regarding handwashing before and after the handwashing education intervention. The findings from this study will aid in enhancing the delivery of handwashing education within the community, ultimately leading to improved perceptions and behaviours regarding handwashing.

## Methods

### Study Population

The interventional study was conducted among 61 Orang Asli students from Sekolah Kebangsaan Pasir Linggi, Gua Musang, Kelantan, aged 7 (Year 1) to 12 years old (Year 6). The study was conducted from June 2024 to August 2024. This school was chosen because it catered not only to students from nearby Orang Asli villages but also to those from other areas in the Gua Musang district, such as Kampung Kuala Koh and Kampung Aring, which are 100 km from the school. Students from distant areas were staying at the school hostel. The broad catchment area enabled the study’s findings to better represent the broader Orang Asli student population in Gua Musang.

In addition to its diverse student base, the school was chosen due to ongoing public health concerns previously reported among the Orang Asli communities in Gua Musang. Several studies and health reports have documented a high prevalence of hygiene-related illnesses in the area, particularly among children. For instance, during the 2019 measles outbreak in Kampung Kuala Koh, which resulted in multiple deaths, healthcare workers noted widespread fungal skin infections and weakened immunity, conditions that are often linked to insufficient hygiene practices (15). Moreover, research focusing on Orang Asli preschool children in Gua Musang reported a high prevalence of malnutrition and soil-transmitted helminth infections, indicating inadequate hygiene practices and poor sanitation in the area (16). Although there have been reports of hygiene issues in the Gua Musang district, there is still limited information about Sekolah Kebangsaan Pasir Linggi, which made it a suitable choice for this handwashing intervention.

### Data Collection

During the first visit, the students were given a set of pre-intervention questionnaires assessing their KAP related to handwashing, and they were subsequently trained in proper handwashing techniques. The handwashing steps are based on recommendations from the World Health Organization and the Centers for Disease Control and Prevention (CDC) Guidelines for Hand Hygiene in Healthcare Settings. After three months, the same questionnaires were administered. As most students had difficulties reading, the questionnaire sessions were conducted through face-to-face interviews during both the pre- and post-intervention visits. The researchers asked the questions verbally and recorded the students’ responses.

### Inclusion and Exclusion Criteria

All students from Years 1 to 6 at Sekolah Kebangsaan Pasir Linggi, Gua Musang, Kelantan, who attended classes during both pre- and post-data collection sessions and whose parents provided consent, were included in the study. Students who were absent on the day of the programme and those who were not well-versed in the Malay language were excluded from the study.

### Sample Size Estimation

There was no information from previous studies about the mean and standard deviation (SD). Therefore, the sample size calculation for all objectives was based on the effect size. The sample size was determined based on two means (paired samples) using G-Power software (version 3.1.9.4) (17). An effect size of 0.4 (between small and medium) was chosen based on Cohen’s *d*, as it reflects a realistic and conservative estimate of the expected change from a school-based hygiene intervention, which typically results in modest behavioural improvements in short-term evaluations (18). This value was deemed suitable due to the educational focus of the intervention and the lack of baseline data available (18, 19). The expected dropout rate was 10%. The calculated sample size was *n* = 52. After accounting for a 10% dropout rate, the estimated sample size was 65 subjects.

### Study Instrument

A validated questionnaire, adapted from Mohamed et al. (12) and Ramli et al. (20), was used to gather information about the students’ KAP regarding handwashing. The first section comprised demographic questions, which included gender, age, parental employment status, whether they lived in the school hostel, whether they had experienced diarrhoea within the last month, and finally, whether they had received training in handwashing before.

Section A of the questionnaire contained six questions related to knowledge of handwashing. Section B covered seven questions about attitudes towards handwashing, and Section C included six questions on handwashing practices. In the knowledge and attitudes sections, respondents were required to select one of five options: strongly disagree, disagree, not sure, agree, and strongly agree. For the practices section, respondents were instructed to select one of three answers: yes, no, or not sure.

### Scoring System

For the knowledge and attitudes sections, a score of 1 was assigned to “strongly disagree,” a score of 2 to “disagree,” a score of 3 to “not sure,” a score of 4 to “agree,” and a score of 5 to “strongly agree.” For the practices section, each “yes” answer received a score of 3, each “not sure” received a score of 2, and each “no” answer received a score of 1.

### Statistical Analysis

Data entry and analysis were performed using IBM SPSS for Windows version 27.0. For the questionnaire, the collected data were entered into the analysis software to verify their accuracy, checking for errors such as missing or duplicate entries. Any inconsistent data were counter-checked and cleaned before the analysis. Categorical data were reported as frequency and percentage. Numerical data were reported as mean and SD if the data were normally distributed. Data were analysed using descriptive statistics and paired *t*-tests to assess the significance of changes. A *P*-value of less than 0.05 was considered significant.

### Ethical Considerations

Written approval from the Human Research Ethics Committee of Universiti Sains Malaysia (JEPeM-USM) was obtained before the commencement of this study. The JEPeM-USM approved this study (Ref: USM/JEPeM/KK/24030285). The purpose and protocol of the study were thoroughly explained to all eligible students, the school, and the students’ parents or guardians. Informed consent was obtained from both the school and the students’ parents or guardians. Verbal consent was also obtained from the students. Privacy of information was strictly preserved.

## Results

The results are presented in the following order: demographic characteristics of participants, detailed findings for the KAP sections, and, finally, overall statistical comparisons by year level.

A total of 61 students participated in this study, comprising 35 females (57.4%) and 26 males (42.6%). Most of the students (18, 29.5%) were from Year 5. The majority of respondents (46, 75.4%) reported experiencing diarrhoea within the last month, and most of them had learnt handwashing techniques before the study (47, 77.0%). In terms of parental employment, nearly all of the students’ fathers (60, 98.4%) were employed, while a smaller proportion of mothers (20, 32.8%) were working, with the majority (41, 67.2%) being unemployed. Additionally, 27 students (44.3%) lived in hostels, while 34 (55.7%) lived at home. Table 1 summarises the demographic data of the respondents.

Tables 2 and 3 demonstrate significant improvements in students’ KAP of handwashing after the education programme, with high scores across all questions. However, in the practices section, there was no change in handwashing after eating, as most students already practised this before the intervention.

Regarding the knowledge section, each question addressed a specific aspect of handwashing, with significant improvements observed across all items. For Question 1, before the intervention, only 8.2% of participants strongly agreed that bacteria could spread from hands to the nose and mouth, while this percentage increased markedly to 72.1% post-intervention. For Question 2, initially, 27.9% of participants agreed that proper handwashing includes six steps, and only 3.3% strongly agreed. After the intervention, the proportion of strong agreement increased to 85.2%, indicating improved understanding of the correct steps. Pre-intervention, only 23% of participants strongly agreed that unwashed hands could cause diarrhoea, whereas this increased significantly to 88.5% post-intervention, showcasing a heightened awareness of the health risks of poor hygiene. The awareness of the role of clean hands in reducing food contamination improved significantly. Prior to the intervention, only 32.8% of participants strongly agreed with this statement. Following the intervention, the proportion of strong agreement increased to 90.2%, indicating a significant improvement in knowledge. For Question 5, while 32.8% of participants agreed and 23.0% strongly agreed that proper handwashing can prevent communicable diseases before the intervention, post-intervention data showed a remarkable increase, with 83.6% strongly agreeing. For Question 6, before the intervention, only 29.5% of participants agreed, and another 29.5% strongly agreed that long nails facilitate bacterial spread. After the intervention, 91.8% of participants strongly agreed, indicating that students gained a stronger understanding of this particular hygiene practice.

In summary, the knowledge section demonstrated substantial improvements across all six questions, with post-intervention strong agreement rates ranging from 72.1% to 91.8%, compared to pre-intervention rates of 3.3% to 32.8%. These findings indicate that the educational programme effectively enhanced students’ understanding of handwashing principles, disease transmission mechanisms, and the importance of proper hygiene practices.

Regarding the attitudes section, scores improved significantly after the intervention, indicating that the handwashing education positively influenced the children’s attitudes towards handwashing practices. For Question 1, before the intervention, 18.0% of participants strongly agreed, while 36.1% agreed that hands should always be kept clean. Following the intervention, the level of strong agreement increased significantly to 93.4%, indicating a heightened awareness of the importance of maintaining clean hands. For Question 2, initially, 18.0% of participants agreed and 41.0% strongly agreed with washing hands with soap after using the toilet. Following the intervention, the proportion of respondents with strong agreement rose to 91.8%, indicating a substantial shift in attitudes towards this critical hygiene practice.

Regarding Question 3, 55.7% of participants strongly agreed and 23.0% agreed that hands should be washed before meals prior to the intervention. Following the intervention, the proportion of strong agreement increased to 93.4%, indicating a deeper understanding of its importance. Regarding Question 4, before the intervention, only 31.1% of participants agreed with this statement, and 45.9% strongly agreed. Following the intervention, the level of strong agreement increased markedly to 91.8%, indicating a significant shift in attitudes. For Question 5, before the intervention, only 45.9% of participants strongly agreed with the habit of washing hands after playing, while 18.0% agreed. After the intervention, strong agreement increased to 88.5%, suggesting a notable improvement in recognising the need to wash hands after playing.

Regarding Question 6, initially, 24.6% of participants strongly agreed and 34.4% agreed with the importance of maintaining short and clean nails. Following the intervention, the proportion of strong agreement rose to 86.9%, indicating a significant shift in attitudes. For Question 7, before the intervention, 34.4% of participants strongly agreed and 18.0% agreed that hand towels should not be shared with others. Following the intervention, the proportion of strong agreement increased to 93.4%, indicating greater awareness of the risks associated with towel sharing.

Overall, the attitudes section revealed marked improvements across all seven questions, with post-intervention strong agreement rates consistently exceeding 86.0%, compared to pre-intervention rates ranging from 18.0% to 55.7%. These results suggest that the intervention successfully shifted students’ attitudes towards recognising the importance of handwashing in various contexts, including before meals, after using the toilet, and after playing.

Practice-related questions demonstrated that the intervention significantly improved students’ actual handwashing behaviour. Almost all questions showed a marked increase in post-intervention scores compared to pre-intervention scores. For Question 1, before the intervention, only 42.6% of participants reported regularly washing their hands with soap after using the toilet, with 57.4% indicating non-compliance. After the intervention, all participants (100.0%) adopted this practice, reflecting a complete behavioural shift. For Question 2, initially, 86.9% of participants washed their hands before eating, while 13.1% did not. After the intervention, adherence improved to 100.0%, demonstrating the effectiveness of the programme in addressing gaps in this practice. Regarding Question 3, prior to the intervention, all participants (100.0%) reported washing their hands after eating, and this trend remained consistent after the intervention, indicating sustained compliance. Regarding Question 4, before the intervention, 44.3% of participants practised handwashing after playing, while 55.7% did not. Post-intervention, this improved substantially, with all participants (100.0%) adopting this behaviour. For Question 5, only 37.7% of participants reported wiping their wet hands until they were dry prior to the intervention, while 60.7% did not. Following the intervention, all participants (100.0%) reported compliance, highlighting a significant improvement in this habit. Regarding Question 6, only 32.8% of participants practised consistent handwashing with soap before the intervention. Post-intervention, all students reported doing so, indicating a significant behavioural improvement.

The practice section demonstrated remarkable behavioural changes, with five out of six questions showing improvement to 100.0% compliance post-intervention, compared to pre-intervention compliance rates ranging from 32.8% to 86.9%. The only exception was handwashing after eating, which maintained 100.0% compliance both before and after the intervention. These findings indicate that the intervention successfully translated knowledge and attitudes into actual behavioural changes.

Table 4 presents the overall scores for KAP before and after the intervention. There was a statistically significant difference in the mean KAP scores before and after the handwashing intervention, with mean differences of 8.672, 7.787, and 5.082, respectively. The 95% confidence intervals (CI) of mean differences in KAP scores before and after the intervention were (7.055, 10.289), (5.635, 9.938), and (4.064, 6.100), respectively, with all scores having *P*-values < 0.001.

Table 5 depicts the comparison of KAP scores before and after the intervention for each year level. Years 2, 3, 4, 5, and 6 showed significant mean differences in knowledge scores between pre- and post-intervention, with mean differences of 19.500, 13.143, 6.167, 4.222, and 9.000, respectively, with all scores having *P*-values < 0.05. However, there was no significant difference in mean knowledge scores between Year 1 before and after the handwashing intervention.

The comparison of attitude scores before and after the intervention showed that Years 2, 3, 4, 5, and 6 had significant mean differences in attitude scores between pre- and post-intervention, with mean differences of 20.667, 14.714, 4.833, 2.722, and 11.214, respectively, with all scores having *P*-values < 0.05. However, there was no significant difference in mean attitude scores between Year 1 before and after the handwashing intervention.

For the comparison of practice scores before and after the intervention, Years 2, 4, 5, and 6 had significant mean differences in practice scores between pre- and post-intervention, with mean differences of 7.000, 4.167, 2.333, and 8.714, respectively, with all the scores having *P*-values < 0.05. However, there was no significant mean difference in practice scores for Year 1 before and after the handwashing intervention. For Year 3, a mean difference in practice scores was observed between the pre-and post-intervention periods; however, this difference was not statistically significant.

Notably, Year 1 students showed no significant improvements in knowledge, attitudes, or practices scores following the intervention, while Year 3 students demonstrated non-significant improvements in practices scores. These findings warrant further discussion regarding the need for age-appropriate intervention strategies, particularly for younger children who may require more tailored educational approaches or extended intervention periods to achieve meaningful behavioural changes.

## Discussion

Proper handwashing is the foundation of personal hygiene, enabling individuals to reduce the spread of infectious diseases, such as diarrhoea and respiratory infections. There have been numerous reports of community-acquired infections caused by multidrug-resistant organisms, such as methicillin-resistant *Staphylococcus aureus* and extended-spectrum beta-lactamase *Escherichia coli* (12). These resistant organisms, which spread through close contact, lead to infections associated with higher mortality, morbidity, healthcare costs, and the need for broad-spectrum antibiotics (12). Thus, conducting school education on handwashing is one of the simplest and most cost-effective methods to reduce the disease burden. Addressing hygiene among Orang Asli kids not only tackles individual problems but also aims to comprehensively resolve health issues within families and the broader society (21).

Orang Asli communities in Malaysia often face limited access to sanitation facilities and clean water, resulting in lower rates of handwashing with soap compared to the general population. A study by Goodson et al. (22) revealed significant differences in nearly all measured variables between Orang Asli and modernised communities in Johor, including household and sanitation facilities, as well as handwashing practices. Approximately 96% of Aboriginal communities relied on tap water, which may have been sourced directly from rivers, local ponds, or public water supplies, while around 86.2% of modernised populations used tap water from municipal water supplies. Water usage among Aboriginal communities was significantly lower than in modernised societies, particularly in terms of mechanical or flush toilets and showering. Regarding handwashing with soap, approximately 18% of the Aboriginal population did not use soap or detergent when washing their hands, compared with 5.8% of respondents from modernised communities. Systematic reviews by Mahmud et al. (8) and Mahmud and Isa (23) also found that Orang Asli populations were at a higher risk of experiencing several tropical diseases and non-communicable diseases than the general population, often due to socioeconomic factors. These studies highlighted the need to improve hygiene education and practices within Orang Asli communities.

The following sections discuss the demographic patterns observed in this study, followed by an analysis of the intervention’s effectiveness across different domains of handwashing behaviour.

According to the demographic data, the number of Year 1 students attending the school was the lowest. This could be attributed to a lack of awareness among Orang Asli parents about the importance of early education (24, 25). Some parents had little or no formal education themselves, making it harder for them to set a positive example for their children (25). Moreover, most parents would ask their children to work and help the family instead of attending school (24). When students were absent from school, their parents tended to remain silent since the children were either assisting them at home or gathering natural products from the forest.

On the other hand, student enrolment was higher in Years 4 to 6 than in Years 1 to 3. This may be due to younger children preferring to learn at their own pace in a more relaxed environment, such as home, in contrast to the structured setting of formal school systems (26). Orang Asli kids had a minimal understanding of the school culture. At home, they were raised to be independent from a very young age, often playing alone, helping with chores, and making their own decisions (27). In contrast, the school environment was structured, time-regulated, competitive, and confined to classrooms. As Orang Asli kids grew older and matured, they became increasingly motivated and inspired to pursue formal education.

Furthermore, as the children grew older, the parents slowly recognised the importance of formal education. A study of Orang Asli awareness of education found that parents were increasingly willing to support their children’s access to better education (28). A study by Mohd Salim et al. (26) also revealed that despite some Orang Asli parents being illiterate, they still did their best to ensure that their children completed their homework and attended school every school day.

Most of the participants had experienced diarrhoea within the past month, which aligns with the findings of studies conducted by Mahmud and Isa (23) and Brito et al. (29). These studies indicated that the prevalence of diarrhoeal diseases was particularly high within Aboriginal communities due to a lack of basic hygiene and sanitation. The frequent occurrence of diarrhoea among Orang Asli kids highlighted the public health problems in this community and the need for better hygiene and sanitation practices.

Over half of the participants had been previously taught proper handwashing techniques, likely due to public health initiatives during the COVID-19 pandemic. During that time, officers from the Ministry of Health Malaysia visited schools and kindergartens in Gua Musang to teach proper handwashing methods. However, many students did not consistently practice proper handwashing, suggesting a gap between knowledge and behaviour.

Overall, the results showed a significant increase in handwashing KAP among participants (*P* < 0.05), indicating that the intervention was effective. The handwashing intervention was suitable for school-age children in rural areas of Malaysia and could also benefit other rural communities in India and Africa. As previously discussed, Orang Asli kids in Malaysia face challenges related to sanitation and water access similar to those experienced by Aboriginal kids in India (30) and Africa (31). These shared environmental and infrastructural barriers underscore the potential transferability of effective handwashing interventions across diverse Aboriginal communities globally.

Handwashing education has effectively improved hygiene in Aboriginal communities in other countries by increasing children’s KAP of handwashing. Our study findings were supported by Umwangange (32), which found that handwashing health education effectively enhanced schoolchildren’s knowledge and skills in both urban and rural public primary schools in Rwanda, Africa (*P* < 0.05). Similarly, a study in South India by Shrestha and Angolkar (33) revealed significant increases in knowledge and practice scores of schoolchildren following a health education intervention (*P* < 0.05). Siwach (34) also reported significant improvements in knowledge and practices after a health education intervention in Panipat, India. Additionally, our findings align with a study conducted by Aiello et al. (35) in the USA, where optimal handwashing practices increased significantly in the control group (*P* < 0.05). A systematic review by Willmott et al. (36) strongly suggested that handwashing interventions had a positive effect on children’s handwashing knowledge, attitudes, and behaviours. These studies highlighted the effectiveness of targeted handwashing education in enhancing public health outcomes in Aboriginal communities worldwide.

However, handwashing education was less effective for Year 1 kids. There was no significant difference in their knowledge and attitude scores before and after the intervention. This may be attributed to a ceiling effect, as they had already received handwashing education from the Ministry of Health in 2023 during their preschool year. During the first visit, many Year 1 children still demonstrated high scores, indicating that they had retained knowledge from their previous education. After the three-month interval, while their knowledge and attitude scores showed no significant change, their behavioural practices remained strong, as evidenced by their consistent “Yes” responses to all practice-related questions. This suggests that while cognitive retention may be limited in younger children, established behavioural habits can persist.

In contrast, students from Years 2 to 6 took the handwashing education more seriously. They understood better, were eager to learn, and remembered what had been taught, resulting in significant improvements in their KAP scores before and after the intervention. A study by Badinlou et al. (37) found that older children were better at using memory strategies to encode and retrieve information than younger children. Badinlou et al. (37) also found that older children required less mental effort to perform cognitive tasks, thereby facilitating easier activation and retrieval of stored information. The study supported the idea that older children have advantages in memory and cognitive processing compared to younger children.

The post-intervention scores for all knowledge-related questions were significantly higher than the pre-intervention scores (*P* < 0.05). Before the handwashing education, Questions 1 and 2 had lower mean scores compared to other questions. Question 1 focused on the understanding of disease transmission, specifically the spread of bacteria from the hands to the nose and mouth, which is a critical pathway for infections such as respiratory diseases. Question 2 evaluated knowledge of proper handwashing techniques, which include six essential steps to remove germs effectively. The low pre-intervention scores for these questions suggested that the Orang Asli kids had limited knowledge of both disease transmission and correct handwashing steps. After the educational programme, these two questions showed a significant increase in scores. These findings were consistent with a study by Kothari et al. (38), which showed that before the intervention, only 3.5% of schoolchildren in the urban area of Pune, Maharashtra, were aware of the correct handwashing technique, whereas awareness increased to 90.7% afterwards. Similarly, a study on primary schoolchildren in rural Malawi revealed a poor understanding of disease transmission principles, aligning with our results (39). The limited knowledge among Orang Asli kids may be due to inadequate health education, limited exposure to hygiene practices, and socioeconomic barriers that restrict access to health information (40). Education plays a key role in increasing knowledge, shaping attitudes, and enhancing self-efficacy – all of which are essential for improving handwashing behaviour.

Following the intervention, scores on all attitude-related questions were significantly higher than pre-intervention scores (*P* < 0.05). Our findings indicated that the mean score for Question 3, which asked whether the children washed their hands after eating, was the highest among all questions before the intervention. This suggested that the practice of washing hands after meals was already a well-established habit among the Orang Asli kids. This finding aligns with a cross-sectional study in which 90.2% of students demonstrated a positive attitude towards handwashing before meals (41). Attitudes can be shaped and transformed through repetitive activities, which are often instilled in children by their parents (41). Such behavioural patterns gradually become ingrained and significantly impact individual behaviour.

The scores for all practice-related questions were significantly higher after the intervention compared to before (*P* < 0.05). No difference in mean score was observed in handwashing practices after eating. Similarly, the pre-intervention score of handwashing practices before eating was higher compared to other practice-related questions. This was because the kids ate with their bare hands. A study revealed that most people in Orang Asli communities in Peninsular Malaysia had a habit of eating with their hands (98.4%) (42). Therefore, they practised washing their hands before and after eating, even without handwashing education. However, the mean practices score for handwashing with soap among Orang Asli kids was the lowest of all practices-related questions before the handwashing education intervention. This finding was consistent with a study by Al-Delaimy et al. (43), which reported that only 18% of Orang Asli schoolchildren in Pahang, Malaysia, practised handwashing with soap before the intervention. The reason behind this may be limited access to soap at certain times. Practice is the actual application of knowledge and attitudes in life. Effective hygiene practices, such as regular handwashing with soap, directly impact health outcomes by reducing the spread of infections and diseases. Consistent practices ensure that the benefits of hygiene knowledge and positive attitudes are realised in daily life.

These findings collectively demonstrate the multifaceted impact of the handwashing education programme on KAP among Orang Asli kids. A strength of our study was the use of direct guidance and visual aids during the handwashing education, which helped engage the students. The participants, from Years 1 to 6, were easily motivated to adopt healthy hygiene habits, such as regular handwashing. However, a limitation of the study was that it was conducted in a single school in a low socioeconomic area, so the findings may not be generalised to other settings.

To improve outcomes, schools should ensure the availability of water and soap to promote effective handwashing practices. Health visitors, community health nurses, and school teachers should organise health campaigns to raise awareness among Aboriginal kids and families. School health nurses can also run health programmes highlighting the importance of handwashing to help reduce school absenteeism.

While the intervention aimed to improve hygiene practices among students, several potential confounding factors may have influenced the study’s outcomes. First, data on parental hygiene practices, which are known to significantly shape children’s hygiene behaviour, were not collected. Prior studies have shown that parental modelling plays a key role in influencing children’s personal hygiene habits; however, these may not be appropriate for Orang Asli parents (44). Second, although our intervention focused on handwashing education, it is essential to acknowledge that the Ministry of Health conducted hygiene promotion activities, including handwashing campaigns, in many schools during the COVID-19 pandemic period. Sekolah Kebangsaan Pasir Linggi was among the schools visited for such initiatives. This prior exposure could have contributed to baseline hygiene awareness and practices among students, potentially reducing the measurable impact of the current intervention. Future research should collect baseline data on parental hygiene practices and control for prior exposure to hygiene education programmes to better isolate the intervention’s independent effects.

## Conclusion

In conclusion, our handwashing education programme significantly enhanced the KAP of handwashing among Orang Asli kids. This improvement highlighted the effectiveness of targeted educational interventions in promoting better hygiene practices. However, the study revealed that Year 1 students demonstrated lower levels of knowledge and attitudes towards handwashing compared to older students. This suggested a need for future interventions to place greater emphasis on younger children, ensuring that they not only understand and memorise proper handwashing techniques but also integrate these practices into their daily routines. These findings address a critical gap in the literature regarding handwashing education in Malaysian Aboriginal communities and provide evidence-based guidance for designing culturally appropriate hygiene interventions in similar contexts.

Given the unique challenges faced by Orang Asli communities, including limited access to clean water and hygiene resources, as well as socioeconomic and cultural factors, it is crucial to tailor educational efforts to address these specific barriers. Since handwashing is one of the most effective and affordable ways to prevent infectious diseases, such as diarrhoea and respiratory infections, which are prevalent in these communities, efforts must be carefully designed to maximise impact. Future programmes should incorporate age-appropriate strategies and resources specifically targeted to younger students. This may include more hands-on activities and frequent repetition to reinforce the importance of good hygiene practices. Consistent handwashing with soap significantly reduces the transmission of harmful pathogens, protecting both individual and community health. Strengthening handwashing practices among young children is crucial in breaking the chain of infection, promoting healthier environments, and reducing school absenteeism due to preventable illnesses. The lessons learnt from this intervention have broader implications for hygiene education in vulnerable communities globally, particularly in settings where cultural sensitivity and resource limitations must be carefully considered in programme design. By prioritising age-appropriate education and addressing contextual barriers, handwashing interventions can achieve sustained behavioural change, ultimately improving health outcomes and reducing disease burden in vulnerable communities.

## Figures and Tables

**Table 1:** Demographic data of respondents (*n* = 61)

Variables	Frequency (%)
**Year**	
1	4 (6.6)
2	6 (9.8)
3	7 (11.5)
4	12 (19.7)
5	18 (29.5)
6	14 (23.0)
**Gender**	
Female	35 (57.4)
Male	26 (42.6)
**Father working**	
Yes	60 (98.4)
No	1 (1.6)
**Mother working**	
Yes	20 (32.8)
No	41 (67.2)
**Live in hostel**	
Yes	27 (44.3)
No	34 (55.7)
**Diarrhoea experience within last month**	
Yes	46 (75.4)
No	15 (24.6)
**Learnt hand wash before**	
Yes	47 (77.0)
No	14 (23.0)

**Table 2:** Comparison of pre- and post-intervention knowledge and attitude on hand washing among participants (*n* = 61)

Questions	Frequency (%)									

	Pre					Post				

	Strongly disagree	Disagree	Not sure	Agree	Strongly agree	Strongly disagree	Disagree	Not sure	Agree	Strongly agree
**Knowledge**										

Bacteria may spread from hands to the nose and mouth	14 (23.0)	4 (6.6)	32 (52.5)	6 (9.8)	5 (8.2)	3 (4.9)	0 (0.0)	4 (6.6)	10 (16.4)	44 (72.1)
The correct techniques of hand washing have six steps	12 (19.7)	3 (4.9)	27 (44.3)	17 (27.9)	2 (3.3)	3 (4.9)	1 (1.6)	2 (3.3)	3 (4.9)	52 (85.2)
Unwashed hands can cause diarrhoea	13 (21.3)	1 (1.6)	14 (23.0)	19 (31.1)	14 (23.0)	4 (6.6)	0 (0.0)	0 (0.0)	3 (4.9)	54 (88.5)
Clean hands may reduce food contamination	12 (19.7)	0 (0.0)	9 (14.8)	20 (32.8)	20 (32.8)	2 (3.3)	0 (0.0)	0 (0.0)	4 (6.6)	55 (90.2)
Proper hand washing can prevent infectious diseases	13 (21.3)	0 (0.0)	14 (23.0)	20 (32.8)	14 (23.0)	2 (3.3)	0 (0.0)	5 (8.2)	3 (4.9)	51 (83.6)
The bacteria can spread easily if we keep long nails	11 (18.0)	0 (0.0)	14 (23.0)	18 (29.5)	18 (29.5)	2 (3.3)	0 (0.0)	0 (0.0)	3 (4.9)	56 (91.8)

**Attitude**										

We must always keep the hands clean	11 (18.0)	0 (0.0)	17 (27.9)	22 (36.1)	11 (18.0)	2 (3.3)	0 (0.0)	0 (0.0)	2 (3.3)	57 (93.4)
We need to wash hands with soap after going to the toilet	6 (9.8)	4 (6.6)	15 (24.6)	11 (18.0)	25 (41.0)	2 (3.3)	0 (0.0)	0 (0.0)	3 (4.9)	56 (91.8)
We need to wash hands before eating	2 (3.3)	0 (0.0)	11 (18.0)	14 (23.0)	34 (55.7)	2 (3.3)	0 (0.0)	0 (0.0)	2 (3.3)	57 (93.4)
We need to wash hands after eating	14 (23.0)	0 (0.0)	0 (0.0)	19 (31.1)	28 (45.9)	2 (3.3)	0 (0.0)	0 (0.0)	3 (4.9)	56 (91.8)
We need to wash hands after playing	10 (16.4)	0 (0.0)	12 (19.7)	11 (18.0)	28 (45.9)	2 (3.3)	0 (0.0)	0 (0.0)	5 (8.2)	54 (88.5)
We have to keep nails short and clean	10 (16.4)	2 (3.3)	13 (21.3)	21 (34.4)	15 (24.6)	2 (3.3)	2 (3.3)	2 (3.3)	2 (3.3)	53 (86.9)
Hand towels should not be shared to	12 (19.7)	3 (4.9)	14 (23.0)	11 (18.0)	21 (34.4)	2 (3.3)	0 (0.0)	0 (0.0)	2 (3.3)	57 (93.4)

**Table 3:** Comparison of pre- and post-intervention practices on hand hygiene among participants (*n* = 61)

Questions	Frequency (%)					

	Pre			Post		

	No	Not sure	Yes	No	Not sure	Yes
**Practice**						

I wash my hands with soap after going to the toilet	35 (57.4)	0 (0.0)	26 (42.6)	0 (0.0)	0 (0.0)	61 (100.0)
I wash my hands before eating	8 (13.1)	0 (0.0)	53 (86.9)	0 (0.0)	0 (0.0)	61 (100.0)
I wash my hands after eating	0 (0.0)	0 (0.0)	61 (100.0)	0 (0.0)	0 (0.0)	61 (100.0)
I wash my hands after playing	34 (55.7)	0 (0.0)	27 (44.3)	0 (0.0)	0 (0.0)	61 (100.0)
I wipe my wet hands until dry	37 (60.7)	1 (1.6)	23 (37.7)	0 (0.0)	0 (0.0)	61 (100.0)
Washing hands with soap is my practice	40 (65.6)	1 (1.6)	20 (32.8)	0 (0.0)	0 (0.0)	61 (100.0)

**Table 4:** Comparison of overall scores between pre- and post-intervention (*n* = 61)

Variable	Mean (SD)		Mean difference (95% CI)	*t*-statistic (df)	*P*-value^a^

	Pre	Post			
Knowledge	19.44 (6.85)	28.11 (4.48)	8.672 (7.055, 10.289)	10.73 (60)	< 0.001
Attitude	25.82 (7.08)	33.61 (5.17)	7.787 (5.635, 9.938)	7.24 (60)	< 0.001
Practice	6.92 (3.98)	12.00 (0.00)	5.082 (4.064, 6.100)	9.98 (60)	< 0.001

a = Paired *t*-test

**Table 5:** Comparison of knowledge, attitude and practice scores between pre- and post-intervention by year (*n* = 61)

Year	Mean (SD)		Mean difference (95% CI)	*t*-statistic (df)	*P*-value^a^

	Pre	Post			
**Knowledge**					

1	6.00 (0.00)	17.00 (12.70)	11.000 (−9.211, 31.211)	1.73 (3)	0.182
2	8.83 (3.60)	28.33 (3.20)	19.500 (15.880, 23.120)	13.85 (5)	< 0.001
3	16.29 (8.50)	29.43 (1.51)	13.143 (5.621, 20.664)	4.28 (6)	0.005
4	23.00 (0.85)	29.17 (1.40)	6.167 (5.361, 6.972)	16.86 (11)	< 0.001
5	25.06 (2.18)	29.28 (1.93)	4.222 (2.962, 5.482)	7.07 (17)	< 0.001
6	19.14 (1.66)	28.14 (1.35)	9.000 (8.183, 9.817)	23.81 (13)	< 0.001

**Attitude**					

1	25.00 (2.31)	21.00 (16.17)	−4.000 (−26.049, 18.049)	−0.58 (3)	0.604
2	14.33 (4.68)	35.00 (0.00)	20.667 (15.759, 25.574)	10.83 (5)	< 0.001
3	20.29 (10.75)	35.00 (0.00)	14.714 (4.772, 24.657)	3.62 (6)	0.011
4	30.17 (2.04)	35.00 (0.00)	4.833 (3.539, 6.128)	8.22 (11)	< 0.001
5	31.50 (3.13)	34.22 (2.26)	2.722 (1.287, 4.157)	4.00 (17)	0.001
6	22.71 (1.44)	33.93 (1.69)	11.214 (10.078, 12.351)	21.32 (13)	< 0.001

**Practice**					

1	4.00 (0.00)	12.00 (0.00)	0.000 (0.000)	0.00 (0)	0.000
2	5.00 (2.45)	12.00 (0.00)	7.000 (4.429, 9.571)	7.00 (5)	0.001
3	8.86 (3.44)	12.00 (0.00)	3.143 (−0.035, 6.321)	2.42 (6)	0.052
4	7.83 (4.04)	12.00 (0.00)	4.167 (1.599, 6.734)	3.57 (11)	0.004
5	9.67 (3.65)	12.00 (0.00)	2.333 (0.520, 4.147)	2.72 (17)	0.015
6	3.29 (1.49)	12.00 (0.00)	8.714 (7.854, 9.575)	21.89 (13)	< 0.001

a =Paired *t*-test
